# Conjunctival Necrosis due to Subconjunctival Methylprednisolone (Depo-Medrol™) Acetate Injection

**DOI:** 10.4103/0974-9233.71587

**Published:** 2010

**Authors:** L. M. van Zyl, J. J. Hill

**Affiliations:** 10 D Molteno Road, Claremont 7732, Cape Town, South Africa; 1 Department of Ophthalmology, Groote Schuur Hospital, University of Cape Town, Cape Town 8000, South Africa

**Keywords:** Conjunctiva, Methylprednisolone, Necrosis

## Abstract

We report a case of conjunctival necrosis due to subconjunctival methylprednisolone (Depo-Medrol™) acetate injection after phacoemulsification surgery. This case report highlights a serious complication of the inadvertent use of methylprednisolone as a subconjunctival agent. To report a case of conjunctival necrosis due to subconjunctival methylprednisolone (Depo-Medrol™) acetate injection after phacoemulsification. Case report a single case presenting to a tertiary ophthalmic unit. An 82-year-old patient underwent uncomplicated phacoemulsification in the right eye. Postoperatively, she was given a subconjunctival injection of methylprednisolone. Two weeks later, she presented with a painful ulcerated lesion of the conjunctiva proximal to the injection site. The ulcerated lesion was surgically excised and she made a complete recovery. In this reported case, methylprednisolone was used in error with significant resultant morbidity. This preparation is not registered for the off label use in ophthalmology, and this case report highlights the danger of its inadvertent use as a subconjuctival agent.

## INTRODUCTION

Subconctival and subtenon injections of corticosteroid are used for a variety of disorders. These include routine postoperative use following anterior segment surgery such as cataract extraction, the treatment of anterior uveitis, and as a deeper subtenon site for posterior inflammatory disease. Usually, a water-soluble preparation of corticosteroid is used and its duration of effect is relatively short. For posterior diseases, it may be necessary to use a longer acting preparation. In this case report, we present a rarely reported complication of using a subconjunctival injection of methylprednisolone (Depo-Medrol™) acetate.

## CASE REPORT

An 82-year-old woman underwent uncomplicated phacoemulsification and intraocular lens implantation of the right eye at a secondary level hospital in Cape Town, South Africa. The standard routine at this hospital is to administer a subconjunctival injection of gentamycin 25 mg and betamethasone 4 mg at the end of surgery. At the time of surgery, since betamethasone was not available, methylprednisolone (Depo-Medrol™) acetate was substituted. The site of the injection was the upper bulbar conjunctiva. She presented 2 weeks later to our hospital complaining of pain and redness at the site of the injection.

**Figure 1:**
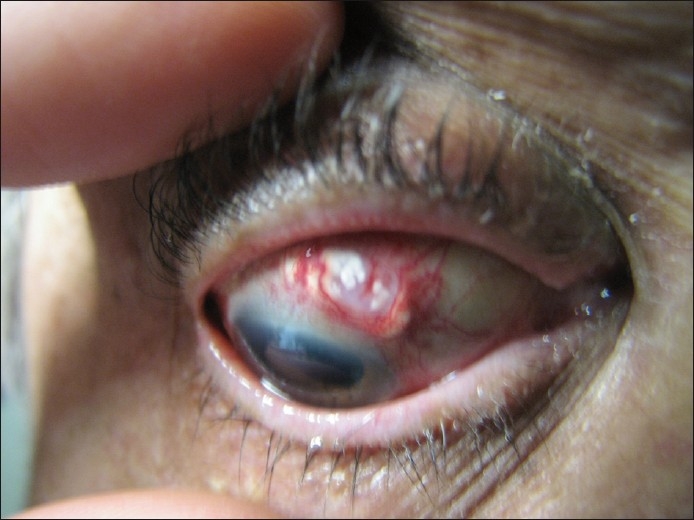
Ulcerating lesion at presentation, superior to the limbus with white precipitates

**Figure 2:**
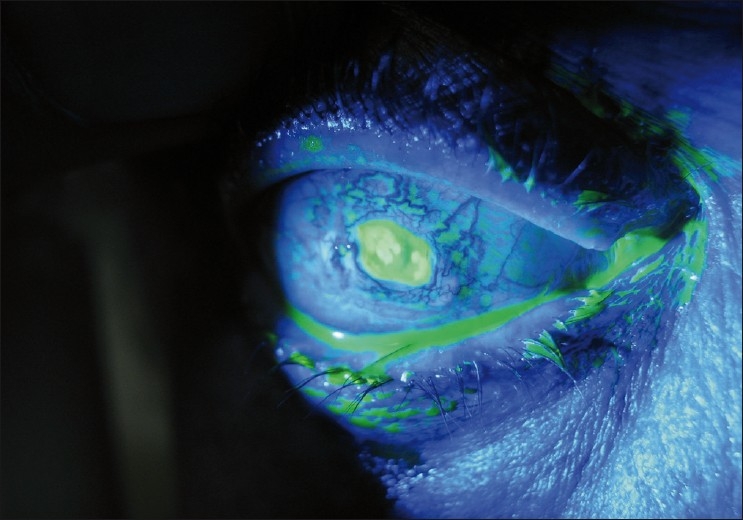
Ulcerating lesion at presentation, staining with fluorescein

The uncorrected visual acuity in the right eye at the time of examination was 6/12. The conjunctiva of the right eye was injected, with an 8 mm by 3.8 mm ulcerating lesion superior to the limbus [Figure 1] which stained with fluorescein [Figure 2], and contained white precipitates at the base of the ulcer. The cornea was clear and the cataract surgical wound was healthy. The anterior chamber was quiet, with no cells or flare. The intraocular lens was well placed in the capsular bag, and the posterior capsule was clear. The vitreous was clear with a posterior vitreous detachment. The macula and peripheral retina were normal. The optic disc was normal, and the intraocular pressure was 20 mmHg.

A conjunctival scrape yielded no microorganisms on microscopy and no growth on culture. The ulcerating lesion was excised, including the white precipitate at the base of the ulcer. Healthy conjunctiva was reflected and sutured to the limbus with Vicryl™ 8.0. Postexcision, the patient was instructed to instil dexamethasone and chloramphenicol drops every 2 hours for 10 days. Histology of the excised lesion confirmed conjunctival and subconjunctival necrosis with inflammatory cell infiltrate and white necrotic tissue. The patient has subsequently been symptom free with a corrected visual acuity of 6/6, One month post excision at her 1-month follow-up appointment.

## DISCUSSION

This is the fourth reported case of conjunctival necrosis following a subconjunctival injection of methylprednisolone. It is the first reported case after phacoemulsification and IOL implantation. Kim and colleagues described the first case in a patient who received several injections for “ocular inflammation.”^1^ Zamir and Pe’er reported a patient who developed conjunctival necrosis after receiving a subconjunctival methyl-prednisolone injection following extracapsular cataract extraction.^2^ Similar to our case, the patient developed symptoms within 2 weeks, and there was a complete recovery after excision of necrotic tissue. Güngor and colleagues reported a case of conjunctival necrosis after a subconjunctival injection of betamethasone following a penetrating keratoplasty.^3^

Methylprednisolone is not registered for off label use in ophthalmology. It is registered as an anti-inflammatory agent for use in rheumatology and in dermatology. It is also registered for systemic control of inflammatory conditions such as ulcerative colitis. The manufacturer warns that incorrect administration can lead to residue and slough at the injection site, as in our case. This report confirms that methylprednisolone should be used with caution as a subconjunctival medication.
